# Worsening Ileal Stenosis Caused by Multiple Ectopic Gastric Mucosa Lesions in an Elderly Patient: A Novel Case with a Long Observation Period

**DOI:** 10.70352/scrj.cr.24-0120

**Published:** 2025-05-17

**Authors:** Ryozan Naito, Etsuko Hisanaga, Ikuma Shioi, Nobuhiro Hosoi, Takayoshi Watanabe, Yuta Shibasaki, Nobuhiro Nakazawa, Katsuya Osone, Takuhisa Okada, Takuya Shiraishi, Akihiko Sano, Makoto Sakai, Hiroomi Ogawa, Makoto Sohda, Yoshihiro Ohno, Ken Shirabe, Hiroshi Saeki

**Affiliations:** 1 Department of General Surgical Science, Gunma University Graduate School of Medicine, Maebashi, Gunma, Japan; 2 Department of Pathology, Gunma University Hospital, Maebashi, Gunma, Japan; 3 Department of Pathology, Tone Central Hospital, Numata, Gunma, Japan

**Keywords:** ileal stenosis, ectopic gastric mucosa, bowel obstruction

## Abstract

**INTRODUCTION:**

Ectopic gastric mucosa (EGM) is a hyperplastic primitive gut epithelium found in tissues other than the stomach. EGM in the small intestine, distal to the ligament of Treitz (EGMdT), is uncommon. EGMdT without congenital anomalies has rarely been reported. Most reported cases are diagnosed in youth, with a single lesion and urgent symptoms requiring emergency surgery. Herein, we report a unique case of multiple EGMdTs without congenital anomalies that caused progressive ileal stenosis in an elderly patient with an observation period of 11 years.

**CASE PRESENTATION:**

The patient was a 77-year-old man. On a medical exam 11 years before arrival at our hospital, the patient was diagnosed with EGMdT in the terminal ileum without stenosis. Five years after the initial diagnosis of EGMdT, the patient experienced his first episode of bowel obstruction, which was treated conservatively. The patient experienced three episodes of bowel obstruction, all of which were treated conservatively. The patient was then referred to our hospital for further examination and treatment. Colonoscopy at our hospital showed an EGMdT that protruded from the Bauhin’s valve. Double-balloon colonoscopy revealed a lymph follicle-like elevation 20 cm from the Bauhin’s valve with obvious stenosis. No malignancies were found on lesion biopsy. The fluoroscopic gastrointestinal series showed five lesions on the oral side of the Bauhin’s valve. Based on these findings, the patient’s symptoms were considered as the outcome of ileal stenosis caused by multiple EGMdTs; therefore, surgical resection was recommended. Laparoscopic resection of the lesion was performed. Intraoperative findings were not suspicious of malignancy. There were five erosions in the resected specimen, and all lesions were diagnosed as EGMdTs without malignancies. The patient was discharged on the 7th postoperative day without any complications. The patient had no recurrent symptoms after discharge.

**CONCLUSIONS:**

Based on the present case, we recommend that clinicians consider surgical resection for symptomatic EGMdT, even without malignancy, and screen for multiple lesions to avoid residual EGMdT.

## Abbreviations

EGM
: ectopic gastric mucosa
EGMdT
: ectopic gastric mucosa distal from the ligament of Treitz
FDG
: ^18^F-fluorodeoxyglucose
MUC
: mucin core protein

## INTRODUCTION

EGM is a hyperplastic primitive gut epithelium found in tissues other than the stomach.^1)^ EGM in the small intestine, distal to the ligament of Treitz (EGMdT), is uncommon.^1)^ Cases associated with congenital anomalies, such as Meckel’s diverticulum^2)^ and intestinal duplication,^3)^ have been reported; however, EGMdT without these abnormalities has rarely been reported.^4)^ Most reported cases are diagnosed in youth, with a single lesion and urgent symptoms requiring emergency surgery. Herein, we report a unique case of multiple EGMdTs without congenital anomalies that caused progressive ileal stenosis in an elderly patient with an observation period of 11 years.

## CASE PRESENTATION

**Fig. 1:**
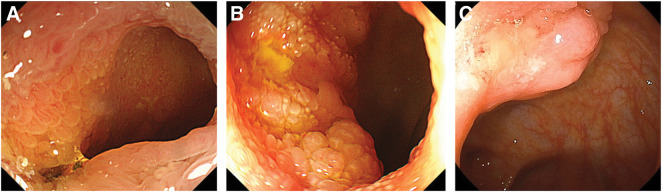
Colonoscopy results. (**A**) First diagnosis of ectopic gastric mucosa. (**B**) Six years after the first diagnosis. Worsening of the ectopic gastric mucosa in the terminal ileum is observed. (**C**) After admission to our hospital. A more aggravated lesion protruding from the Bauhin’s valve is observed.

**Fig. 2:**
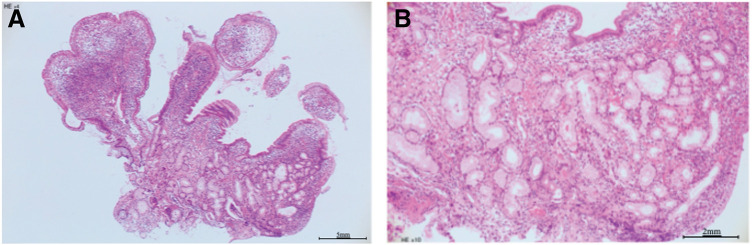
Microscopic findings of the first biopsy. (**A**) Hematoxylin and eosin staining in a low-power field. (**B**) Hematoxylin and eosin staining in a high-power field.

**Fig. 3:**
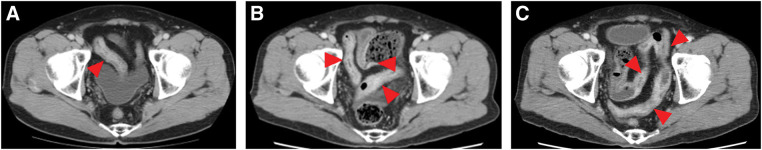
Computed tomography findings. (**A**) Initial diagnosis. Slight wall thickening in the small bowel is observed (arrow). (**B**) Five years after the first diagnosis. Wall thickening has worsened compared with that at first diagnosis (arrows). (**C**) After admission to our hospital. Extension of the stenotic lesions is observed (arrows).

The patient was a 77-year-old man with a medical history of hepatitis C which sustained a complete virological response. On a medical exam 11 years before arrival at our hospital, the patient was diagnosed with EGMdT in the terminal ileum without stenosis (**Figs. 1A**, **2A**, **2B**). CT revealed slight wall thickening in the small bowel (**Fig. 3A**). Five years after the initial diagnosis of EGMdT, the patient experienced their first episode of bowel obstruction, which was treated conservatively. CT revealed worsening wall thickening in the small bowel (**Fig. 3B**). Colonoscopy performed the following year revealed worsening of the EGMdT with mild stenosis (**Fig. 1B**). The patient experienced three episodes of bowel obstruction, all of which were treated conservatively. The patient was then referred to our hospital for further examination and treatment of bowel obstruction.

**Fig. 4:**
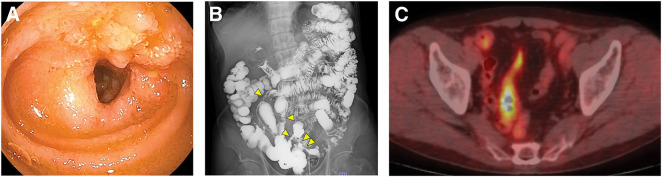
Further examination findings in our hospital. (**A**) Double-balloon endoscopic findings showing a lymph follicle-like elevation 20 cm from the Bauhin’s valve. (**B**) Fluoroscopic gastrointestinal series showing several narrow lesions on the oral side of the Bauhin’s valve. (**C**) Positron emission tomography-computed tomography showing the accumulation of 18F-fluorodeoxyglucose in the stenotic area.

**Fig. 5:**
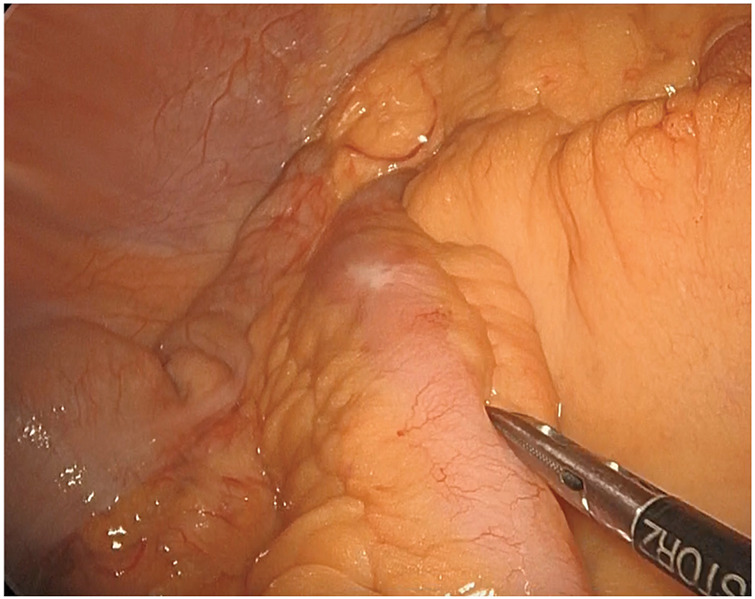
Intraoperative findings showing inflammatory changes in the serosa.

On referral, the patient required oral liquid nutritional support to complement his poor oral intake. Colonoscopy at our hospital showed a rise of EGMdT that protruded from the Bauhin’s valve (**Fig. 1C**). Double-balloon enteroscopy revealed a lymph follicle-like elevation 20 cm from the Bauhin’s valve with obvious stenosis (**Fig. 4A**). No malignancies were found on lesion biopsy. The fluoroscopic gastrointestinal series showed five lesions on the oral side of the Bauhin’s valve (**Fig. 4B**). CT revealed worsening wall thickening in the small bowel with multiple lesions (**Fig. 3C**). Positron emission tomography-CT showed FDG accumulation in a similar area (**Fig. 4C**). Based on these findings, the patient’s symptoms were considered as the outcome of ileal stenosis caused by multiple EGMdTs; therefore, surgical resection was recommended at the board conference. Surgical findings revealed only a small inflammatory change in the serosa within 100 cm of the terminal ileum (**Fig. 5**), which was contradictory to malignancy. The lesion was easily palpable as an elastic soft lesion of the small intestine. The small bowel was carefully examined, which revealed no new lesions. Intraoperative endoscopy was performed through an incision in the small bowel, and residual lesions within the observable range were ruled out. Laparoscopic resection of the lesion without regional lymph node dissection was performed. The patient was discharged on the 7th postoperative day without any complications and had no recurrent bowel obstruction after discharge.

**Fig. 6:**
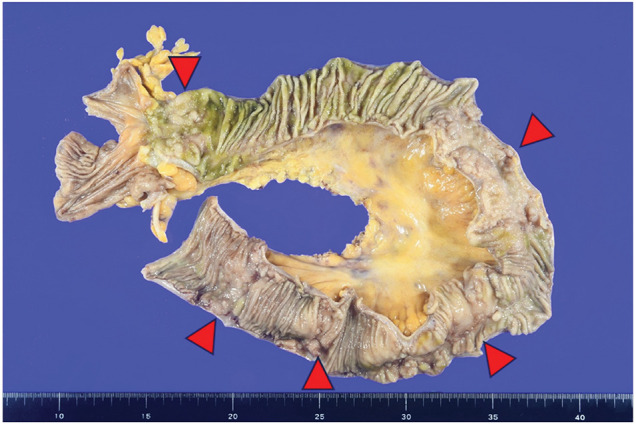
Macroscopic findings of the extracted specimen. Arrows represent five elevated lesions and narrowing of the lumen caused by ectopic gastric mucosa.

**Fig. 7:**
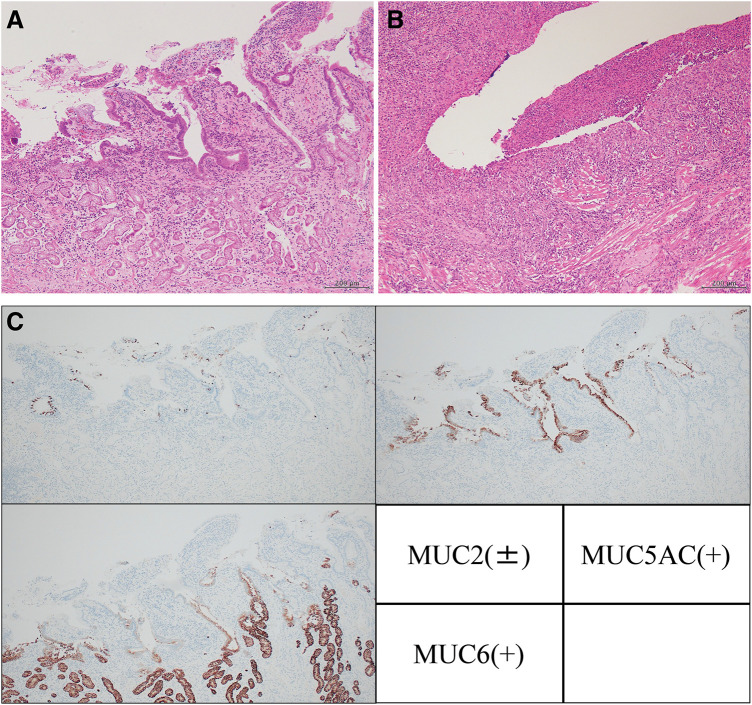
Microscopic findings of the specimen. (**A**) Ectopic gastric mucosal tissue. The surface is covered with gastric glandular epithelium, and pyloric glands can also be seen. (**B**) Active ulcer. Exudate and inflammatory granulation tissue are present, with the tissue corresponding to the ulcer base. (**C**) Immunohistochemical staining results. MUC2: Negative in the gastric glandular epithelium. However, some of the epithelium is positive for intestinal metaplasia and MUC2. MUC5AC: Positive in the gastric glandular epithelium. MUC6: Positive in the pyloric glands. MUC, mucin core protein

On macroscopic examination, the extracted ileum had five soft, partial white-tone elevations and narrowing of the lumen (**Fig. 6**). The other mucosa was normal without any inflammation. Histopathological examination of the raised area revealed ectopic gastric mucosal tissue (**Fig. 7A**). In addition, an active ulcer (**Fig. 7B**) was seen in the vicinity. The former was covered with glandular epithelium and accompanied by pyloric glands. In the latter, the epithelium was erosive, with exudate and inflammatory granulation tissue, and the tissue image corresponded to the ulcer base. Immunostaining (**Fig. 7C**) of the gastric glandular epithelium showed MUC2 negativity. However, some of the epithelium was intestinal metaplasia-positive and MUC2-positive. In addition, the gastric glandular epithelium and pyloric gland were positive for MUC5AC and MUC6, respectively. There was no evidence of malignancies, Crohn’s disease, or intestinal tuberculosis.

## DISCUSSION

Herein, we report a unique case of worsening ileal stenosis caused by multiple EGMdTs in an elderly patient with an observation period of 11 years. Articles concerning EGMdT without congenital anomalies were identified in PubMed from inception to December 2023 using the following search terms: “ectopic gastric mucosa” and “gastric heterotopia.” All retrieved articles were manually assessed to identify EGMdT cases without congenital anomalies. Consequently, 19 cases were extracted^4–22)^ (**Table 1**). The mean (range) age was 32 (16–77) years, and only one case was more than 75 years old. The main reasons for diagnosis were ulcers,^4)^ gastrointestinal bleeding,^8)^ perforations,^11)^ bowel obstruction,^15)^ and intussusception.^18)^ Most patients with symptomatic EGMdT have severe symptoms that need urgent operation.^4,8,15,18)^ Only five articles included patients with multiple EGMdTs without congenital anomalies.^5,13,16,17,20)^ A case of multiple EGMdTs in an elderly patient is unique, and there have been no cases with a long observation period, as in the present case.

**Table 1:** Summary of previous reports

Year	Author	Age	Discovery opportunity	Single/Multiple	Treatment	Outcome
1974	Nawaz^5)^	43	Gastrointestinal bleeding	Multiple	Resection	Alive
1987	Lodge^6)^	16	Bowel obstruction	Single	Resection	Unknown
1988	Caruso^7)^	46	Bowel obstruction	Single	Resection	Alive
1993	Karakatsanis^8)^	16	Gastrointestinal bleeding	Single	Resection	Alive
2007	Omotosho^9)^	17	Bowel obstruction	Single	Resection	Alive
2007	Hammers^10)^	16	Bowel intussusception	Single	Resection	Alive
2007	Hurley^11)^	77	Bowel obstruction	Single	Resection	Alive
2009	Khan^12)^	36	Bowel obstruction	Single	Resection	Alive
2011	Schaefer^13)^	68	Bowel obstruction	Multiple	Resection	Unknown
2013	Chinnery^14)^	17	Bowel obstruction	Single	Resection	Unknown
2013	Martínez^15)^	21	Bowel obstruction	Single	Resection	Unknown
2015	Tai^16)^	70	Positive fecal occult blood test	Multiple	Observe	Alive
2018	Abu-Zidan^4)^	39	Bowel obstruction	Single	Resection	Alive
2019	Park^17)^	26	CT image abnormality	Multiple	Unknown	Alive
2019	Hazan^18)^	18	Bowel intussusception	Single	Resection	Alive
2019	Ali^19)^	23	Gastrointestinal bleeding	Single	Resection	Alive
2022	Chen^20)^	54	Bowel obstruction	Multiple	Unknown	Alive
2022	Redzepagic^21)^	16	Bowel intussusception	Single	Resection	Alive
2023	McCarthy^22)^	23	Bowel intussusception	Single	Resection	Alive

Schaefer et al. noted that heterotopic gastric mucosa of the small intestine has malignant potential.^13)^ Therefore, careful examination is necessary. In the current case, the site and degree of wall thickening clearly worsened over the long observation period. Therefore, we considered that this case had potential for a malignant lesion. However, the radiologic and endoscopic findings were not typical for malignancy. Also, the FDG PET scan was not useful for ruling out malignancies because physiological FDG uptake is frequently observed in the gastrointestinal tract. As a result, ruling out malignancies in the preoperative examination was difficult in this case. However, the surgical findings were inconsistent with malignant lesions, and therefore simple resection was selected without regional lymph node dissection. The histopathological examination revealed elevations in the EGM lesion with inflammatory granulation tissue and fibrosis without malignant tissue. This finding suggests that EGMdT may cause very slow but progressive stenosis, even in the absence of malignancies. Comparing the surgical specimen with the biopsy specimen obtained 11 years prior, inflammation and fibrosis had worsened. Therefore, it is thought that the ulcer may have been caused by gastric acid secreted from the ectopic gastric mucosa and that lumen narrowing was caused by the cycle of ulcer formation and healing.

In the previous literature,^17,18)^ EGM has been broadly classified into congenital and acquired types. Congenital EGM is caused by abnormal embryological development, whereas acquired EGM results from acquisition secondary to inflammation or disease. In the present case, considering that the number of lesions had not increased and that the surface was covered with gastric glandular epithelium along with the presence of pyloric glands, it is highly likely that this represents congenital EGM. Regarding the cause of exacerbation, although this patient is elderly, we speculated that EGMdT progressively worsened over time due to inflammation caused by acid produced by the lesion itself.

There are no clear criteria regarding the indications for surgical treatment of EGMdT owing to its rarity. Of the 19 patients with symptoms listed in **Table 1**, 16 underwent surgical resection. The postoperative courses of all patients were uneventful, and no symptom recurrence was observed. However, the treatment strategies and outcomes in symptomatic patients after conservative therapy remain unclear. In the present case, the patient was unable to have normal oral intake upon arrival at our hospital due to stenosis caused by EGMdT, which was first diagnosed 11 years ago. As hypothesized above, stenosis caused by EGMdT may progress slowly regardless of malignancy. Therefore, the patient may have avoided long-lasting symptoms by earlier surgical treatment. In addition, multiple EGMdT lesions were observed in the present case. Clinicians must be aware of the possibility of residual EGMdT and thoroughly check for it during pre- and intra-surgical examinations with palpation or intraoperative endoscopy, as was performed in the present case.

A limitation of this case is that we did not consider endoscopic examination of the total small bowel. Since the extent of resection was determined by intraoperative palpation and endoscopy, the risk of small residual lesions remains. It is better to perform a double-balloon endoscopy from the oral side to lower the risk of residual lesions.

## CONCLUSIONS

Herein, we present a unique case of ileal stenosis caused by multiple EGM lesions in an elderly patient. Based on the long observation period of 11 years, EGMdT is considered a lesion that may cause very slow but progressive stenosis, even without malignancies. We recommend that clinicians consider surgical resection for symptomatic EGMdT, even without malignancy, and screen for multiple lesions to avoid residual EGMdT.

## ACKNOWLEDGMENTS

The authors thank Editage (www.editage.com) for the English-language editing.

## DECLARATIONS

### Funding

No funding was received to assist with the preparation of this manuscript.

### Authors’ contributions

RN reported this case and wrote the manuscript.

IS, NH, TW, YS, NN, KO, TO, TS, AS, MSa, HO, and MSo were involved in diagnosis and treatment.

EH and YO gave the histopathological diagnosis.

KS and HS supervised the study.

All authors have read and approved the final manuscript.

All authors agree to take responsibility for all aspects of this research.

### Availability of data and materials

Data sharing is not applicable to this article as no new data were created or analyzed in this study.

### Ethics approval and consent to participate

This case report was written under the ethical standards of the Declaration of Helsinki (as revised in Brazil 2013). Written informed consent was obtained from participants for this article and any accompanying images.

### Consent for publication

The patient provided written informed consent prior to their inclusion in the case report.

### Competing interests

The authors declare no conflicts of interest associated with this manuscript.
